# MYB oncoproteins: emerging players and potential therapeutic targets in human cancer

**DOI:** 10.1038/s41389-021-00309-y

**Published:** 2021-02-26

**Authors:** Ylenia Cicirò, Arturo Sala

**Affiliations:** Department of Life Sciences, Centre for Inflammation Research and Translational Medicine, Brunel University London, UB8 3PH Uxbridge, UK

**Keywords:** Cancer, Oncogenes

## Abstract

MYB transcription factors are highly conserved from plants to vertebrates, indicating that their functions embrace fundamental mechanisms in the biology of cells and organisms. In humans, the *MYB* gene family is composed of three members: *MYB*, *MYBL1* and *MYBL2*, encoding the transcription factors MYB, MYBL1, and MYBL2 (also known as c-MYB, A-MYB, and B-MYB), respectively. A truncated version of MYB, the prototype member of the MYB family, was originally identified as the product of the retroviral oncogene *v-myb*, which causes leukaemia in birds. This led to the hypothesis that aberrant activation of vertebrate MYB could also cause cancer. Despite more than three decades have elapsed since the isolation of v-myb, only recently investigators were able to detect *MYB* genes rearrangements and mutations, smoking gun evidence of the involvement of *MYB* family members in human cancer. In this review, we will highlight studies linking the activity of *MYB* family members to human malignancies and experimental therapeutic interventions tailored for *MYB*-expressing cancers.

## Introduction

Vertebrate *MYB* genes encode transcription factors related to the *v-myb* oncogene, the transforming gene of avian retroviruses causing myelomas and lymphomas in birds^1,2^. AMV was originally identified as a virus that induces a disease in chickens similar to acute myelogenous leukaemia in humans^3^. The *v-myb*^*AMV*^ oncogene product, a 45 kDa protein, was proved to be a truncated version of vertebrate MYB, the 75 kDa product of the proto-oncogene *MYB*, mainly expressed in haematopoietic tissues^4,5^. The *v-myb* oncogene was also found fused to a second oncogene, *v-ets*, in the E26 retrovirus that cause avian erythroblastosis^6^. Invertebrates carry only one *MYB* gene which, from a phylogenetical and functional point of view, is equivalent to vertebrate *MYBL2*, suggesting that this is the most ancient member of the family^7,8^. There is no homologue of the *MYB* gene in nematodes, although distantly related genes, such as *Cdc5* and *SNAPc*, have been identified in *Caenorhabditis elegans*^9,10^.

**Fig. 1:**
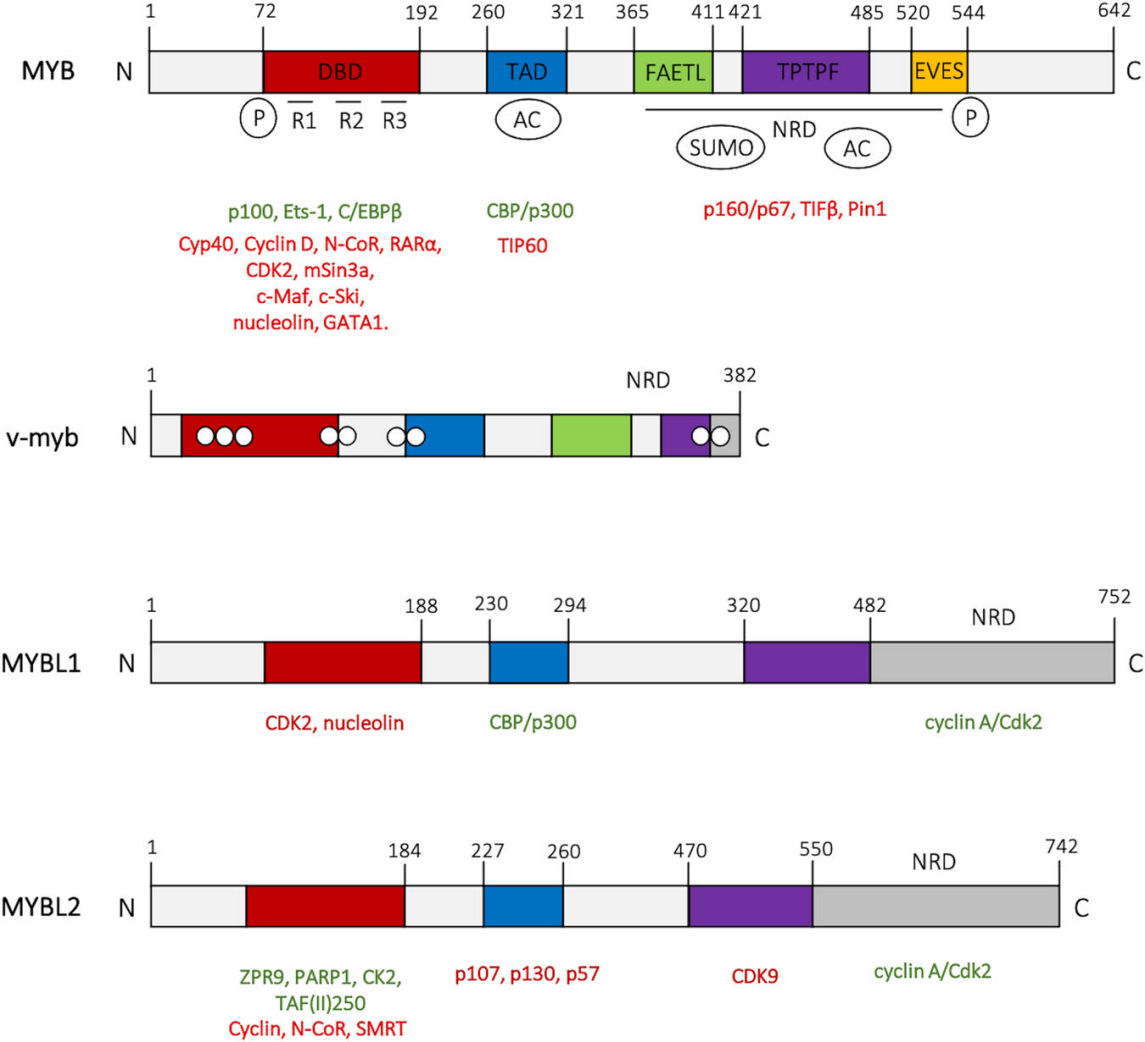
MYB family members’ protein structures. The v-myb DNA-binding domain is equivalent to amino acids 72–192 of MYB, except the introduction of four point mutations (I91N, L106H, V117D, and I181V) and the addition of six amino acids in N-terminal region derived from the retroviral Gag polyprotein^187^. The white dots on AMV v-myb structure indicate point mutations important for the ability of v-myb to transform cells^188^. MYB co-activators are listed in green and the co-repressors are listed in red. The DNA-binding domain (DBD) is comprised of three repeats (R1, R2, and R3). It is the binding site for a number of proteins including p100, PARP, c-Ski, N-CoR, RAR, Cyp40, C/EMPbeta, SMRT, and mSin3A, as depicted; the central transactivation domain (TAD) is the interaction site for CBP/p300; the negative regulatory domain (NRD) extends from the FAETL motif to the EVES peptide sequence (involved in intramolecular and intermolecular protein–protein interactions) and includes the binding sites for p160/p67, Pin1, and TIF1beta^150,189–193^. The post-translational modifications include phosphorylation (P), acetylation (AC), and sumoylation (SUMO)^194–197^.

In humans and other mammals, the transcription factor MYB (encoded by *MYB*) is the prototype member of the family, which includes MYBL1 (encoded by *MYBL1*) and MYBL2 (encoded by *MYBL2*)^11^. Although similar in structure, the different MYB proteins interact with unique co-factors and their expression is often nonoverlapping, suggesting that they might have distinct biological roles (Fig. 1)^12–15^.

## MYB proteins structure and identification of target genes

MYB proteins contain a highly conserved helix-turn-helix (HTH) DNA-binding domain (DBD) at the N-terminus, encompassing three tandem repeated domains of ~50 amino acids containing tryptophan named R1, R2, and R3^16^; a conserved C-terminal negative regulatory domain (NRD); a trans-activating domain (TAD) in the central portion of the protein. The latter includes an acidic region and a heptad leucine-zipper repeat only present in MYB and MYBL1 (Fig. 1)^9^.

All MYB family members recognise and bind the same DNA consensus sequence [PyAAC(G/T)G] to transactivate gene expression. This motif, firstly identified by the Klempnauer group using DNA footprinting assays, is known as the canonical MYB-binding site (MBS)^17^. The sequence was later confirmed to be present, and bound by v-myb, in the promoter region of the first MYB-target gene identified in vertebrates, *mim-1*^18^. With the development of more advanced genomic technologies, different groups attempted the identification of MYB target genes at the global level. The Ness team found the c-MYB protein bound to over 10,000 promoters in the cancer breast cell line MCF-7, and validated known MYB target genes involved in the cell cycle, such as *MYC* and *CCNB1*, or identified new MYB target genes involved in stemness and transcription control such as *JUN, KLF4, NANOG* and *SND1*^19^. Another study by the Gonda lab identified genes regulated by MYB in mouse myeloid progenitor cells. This study not only confirmed that MYB positively regulates promoters of key cell proliferation genes, such as *Myc*, but it can also work as a transcriptional repressor. Indeed, several key regulators of myeloid differentiation such as *Runx1*, *Pu.1*, *Junb* and *Cebp* were strongly suppressed by exogenous expression of *MYB*, suggesting a mechanism used by the transcription factor to suppress differentiation and promote self-renewal^20^. A selection of *MYB* target genes that have been shown to mediate physiological functions in normal or disease contexts is shown in Table 1.

**Table 1:** Selected MYB target genes.

Target gene	Protein	MYB member	References
*ATR*	Ataxia telangiectasia Rad3-related protein	MYB	^43^
*BCL2*	B-cell lymphoma 2	v-Myb, MYBL2,MYB	^137,198,199^
*BIRC5*	Survivin	MYBL2, MYB	^136,200,201^
*CCNA2*	Cyclin A2	MYBL2	^149,202^
*CCNB1*	Cyclin B1	MYB	^19,38,203^
*CCND1*	Cyclin D1	MYBL2	^131,204^
*CCNE1*	Cyclin E1	MYB	^205,206^
*CD34*	Haematopoietic progenitor cell antigen CD34	MYB	^207–209^
*CDK1*	Cyclin-dependent kinase 1	MYBL2, MYB	^42,149,202^
*CDK2*	Cyclin-dependent kinase 2	MYBL2	^210^
*CDK6*	Cyclin-dependent kinase 6	MYB	^211^
*CLU*	Apolipoprotein J/Clusterin	MYBL2	^132,134,212^
*CXCR4*	C-X-C chemokine receptor type 4	v-Myb, MYB	^19,213^
*IGF1R*	Insulin-like growth factor 1 receptor	MYB	^106,107,109,113^
*KIT*	(c-)KIT/CD117	MYB	^119,214,215^
*MIM1*	Mitochondrial import protein 1	v-Myb, MYB	^18,216,217^
*MYC*	(c-)MYC	MYBL2, MYB	^19,20,138,210,218^
*NCAPH*	Non-SMC Condensin I Complex Subunit H	MYBL2	^169^
*PLK1*	Polo-like kinase 1	MYBL2	^157,159,202^
*TAL1*	T-cell acute lymphocytic leukaemia protein 1	MYB	^27^
*VEGF*	Vascular endothelial growth factor	MYB	^118^

The transcriptional activity of MYB proteins is regulated either positively or negatively by co-factors; cellular proteins physically interacting with the different MYB family members are indicated under their protein structures in Fig. 1. Structure–function relationships have been largely inferred by studying the prototype member of the family, MYB (c-MYB). For example, the TAD domain confers transactivating activity to MYB by recruiting CREB-binding domain protein (CBP) and p300^21,22^. The CAAT enhancer-binding protein (C/EBP) family member NF‐M cooperates with MYB in transcriptionally activating the *mim‐1* promoter through an adjacent DNA-binding site and it is also co‐activated by CBP in a Ras‐dependent manner, suggesting that CBP might work by functionally linking MYB and NF-M^22^. Indeed, NF-M has been shown to affect the MYB-C/EBP interaction by disrupting the N-terminal region within the repeat domain R1 (amino acids 47–71), enhancing MYB oncogenic activity^23^.

MYB can cooperate, cross-regulate and compete with other transcription factors, such as members of the C/EBP family, the ETS family, and GATA1^24–26^. Recently, it has been shown that in ALL patients aberrant recruitment of the histone acetyl transferase CBP/p300 by MYB in the enhancer region of the protooncogene *TAL1* occurs via the formation of de novo MYB-binding elements^27^.

## Alterations of *MYB* family genes in human cancer and experimental therapeutic approaches

*MYB* family members are often aberrantly expressed in human cancers, suggesting that they could be important for tumour initiation and/or maintenance. Since MYB proteins are essential for key cellular processes such as growth, differentiation and survival, it is likely that genomic mutations or alterations of gene expression might contribute to oncogenesis. Broadly expressed transcription factors are considered unsuitable therapeutic targets since their inactivation or downregulation could be detrimental to organism homoeostasis. Furthermore, it is inherently difficult to block the interaction of transcription factors with DNA using small molecules. Despite these caveats, therapeutic approaches aiming at inhibiting MYB oncoproteins, or their target genes, in cancer are under investigation in preclinical and clinical studies.

In the following paragraphs, we discuss studies in which *MYB* family members have been implicated in forms of human cancer. We also highlight laboratory experiments, or clinical trials, in which *MYB*, or *MYB*-regulated genes, have been targeted for therapeutic purposes.

### *MYB*

Disruption of *MYB* causes embryonic lethality due to the failure of foetal hepatic haematopoiesis^28^. The key role of the *MYB* gene product in mammalian haematopoiesis is also indicated by its ability to regulate the expression of foetal haemoglobin and requirement for the maturation of T and B lymphocytes^29–32^. Although prevalently expressed in haematopoietic cells, *MYB* expression is detected also in neural tissues, as well as in colonic crypts and breast cells^33–37^.

MYB, similarly to the ubiquitous member of the family MYBL2, regulates cyclin-dependent kinases (Cdks) expression and activity, essential for cell duplication^38,39^. *MYB* autoregulates its own expression and is engaged in positive and negative regulatory loops with cyclins and Cdks, in both the G1 and G2 phases of the cell cycle^38,40–42^.

#### *MYB* alterations in cancer

Genetic mutations and augmented expression of *MYB* have been firstly noted in leukaemic cells, and only relatively recently in solid cancers. Overexpression of wild type *MYB* is insufficient for full transformation of human epithelial cells, supporting the hypothesis that it promotes tumourigenesis only in combination with additional genetic alterations^43^.

The first recurrent genomic rearrangements of the *MYB* locus were evidenced in acute T cell leukaemia, in which *MYB* overexpression is caused by gene duplication or translocation, juxtaposing strong enhancers from other genomic locations^44^. Summarising the information present in literature, it is possible to group *MYB* oncogenic alterations into three classes: overexpression, fusion with partner genes, and ectopic binding of the MYB oncoprotein to enhancer sequences caused by somatic mutations (i.e. *TAL1* enhancer^27^). *MYB* gene amplification and overexpression have been observed in acute myeloid leukaemia (AML), non-Hodgkin lymphoma, colorectal cancer, and breast cancer^5,45–48^. Fusion with partner genes is mainly observed in solid tumours, as discussed in detail in the following sections.

*MYB* genomic alterations have been detected in multiple forms of human cancer, suggesting a causative role. Therefore, numerous studies have been conducted in which inhibition of *MYB*, or of its downstream genes, has been used as a potential therapeutic strategy. Preclinical studies and actionable MYB target genes are summarised in Table 2.

**Table 2:** Preclinical and clinical therapeutic strategies based on inhibition of MYB or actionable MYB-target genes.

Treatment	Target	Cancer type	References
AT7519, BE-09-LN53 (CDKi)	CDKs	ER + BC	^90^
ATRA	MYB	ACC	^111^
Celastrol	MYB-C/EBPβ-p300	AML	^67^
Mebendazol	MYB	AML	^69^
miR-200b/c	EMT markers	ER + BC	^94^
Monensin A	MYB	AML, ACC	^110^
MYBMIM	MYB:CBP/p300	AML	^71^
Naphthol AS-E phosphate	MYB-C/KIX(p300)	Leukaemia	^68^
Plumbagin	MYB/p300	AML	^70^
TetMYB vaccine	MYB	CRC, ACC	^81^
VX-970	ATR	ACC	^43^
Figitumumab	IGFR	ACC	^109^
Linsitinib	IGFR	ACC	^112^

*ATRA* all trans retinoic acid, *ACC* adenoid cystic carcinoma, *AML* acute myeloid leukaemia, *CDKs* cyclin-dependent kinases, *CRC* colorectal cancer, *EMT* epithelial mesenchymal transition, *ER* *+* *BC* oestrogen receptor positive breast cancer.

##### *MYB* and leukaemia

In a cluster of acute lymphoblastic leukaemia (ALL) patients, mutations of the *TAL1* enhancer create ex-novo MYB-binding sites. The leukaemias arising in these patients show *MYB*-dependency consequential to the aberrant activation of the *TAL1* oncogene by MYB^27^. Through genomic screening of an independent set of 107 individuals with T cell ALL (T-ALL) and 12 T-ALL cell lines, Lahortiga et al. detected duplication of *MYB* in 9 of 107 (8.4%) cases and in five different cell lines^49^. The flanking genes *HBS1L* and *AHI1* were duplicated in some patients, but the commonly duplicated region covered only the *MYB* gene. The duplication is associated with a threefold increase in *MYB* expression, and its knockdown initiates T cell differentiation. Thus, *MYB* duplications may be leukaemogenic in a subset of T-ALL patients^49^.

In acute basophilic leukaemia (ABL) the *MYB* locus is fused to another gene encoding the transcription factor GATA1. This rare subtype of acute myeloblastic leukaemia is characterised by the t(X;6)(p11;q23) translocation, leading to decrease or loss of *GATA1* (located on chromosome X) expression^50^. Mice transgenically expressing the *MYB–GATA1* fusion develop myelodysplasia and leukaemia when endogenous, wild-type *GATA1* expression is concurrently downregulated^51^. Ducassou and co-workers showed that the fusion promotes not only haematopoietic progenitor cell self‐renewal, but also induces a bias toward granulocytic differentiation, consequently to sensitisation towards NGF- and IL-33-induced differentiation^52^. The skewing towards basophilic differentiation was confirmed in primary human CD34‐positive stem/progenitor cells, where the basophilic markers CD203c and FcϵRI were activated after *MYB–GATA1* expression. In vivo experiments using NSG mice led to conclusive evidence that basophilic differentiation is a direct consequence of *MYB-GATA1* expression, rather than loss of endogenous *GATA1*^52^. The increased responsiveness to IL-33 could contribute to the leukaemic phenotype, as previously observed in other myeloproliferative malignancies^53^. Thus, *MYB-GATA1* might promote cell growth, self-renewal and leukaemic transformation of basophilic progenitor cells^52^.

A case report described a Philadelphia-negative myeloproliferative neoplasm (Ph-MPN) with an uncommonly rapid leukaemic progression, linked to JAK2^V617F^ mutation. This primary myelofibrosis (PMF)-patient developed a peculiar chromosomal rearrangement resulting in a fusion involving *EWSR1* and *MYB*. There are only a few cases reporting fusion of *EWSR1* in leukaemia, whereas it is common in soft tissue sarcoma^54–56^. *EWSR1* is a FET (FUS, EWS, TAF15) family member whose function is to regulate transcription and mRNA splicing^57^. Therefore, it seems reasonable to speculate that the *EWSR1-MYB* fusion could lead to dysregulated *MYB* transcriptional activity. Indeed, expression of the MYB target gene *BCL2* was deregulated in *EWSR1-MYB* positive PMF, suggesting that molecular alterations involving *MYB* could increase disease risk in PMF patients^58^.

AML is the most common form of acute leukaemia in adults^59^. Although recent advances in genomic characterisations have shed some light on the molecular patterns involved in this cancer, the 5-year survival rate is <70% in children and 35% in adults^60,61^.

AML is a heterogeneous disease, often characterised by the presence of gene fusions or recurrent mutations in a set of driver genes^62^. Genomic rearrangements involving the *MLL* gene, such as *MLL–AF4* t(4;11)(q21;q23); *MLL–AF9*; t(9;11)(p22;q23); *MLL–ENL*; t(11;19)(q23;p13.3); *MLL–AF10* t(10;11)(p12;q23) or *MLL–AF6* t(6;11)(q27;q23) are associated with a very aggressive form of leukaemia^63,64^. *MYB* has been shown to be a key downstream effector of MLL fusion oncoproteins, suggesting that it could be a target for therapeutic interventions^65^. Since, as mentioned before, targeting transcription factors with small molecule inhibitors is difficult, the focus has been directed towards proteins that work as co-activators in the MYB network. p300 is a MYB transcriptional co-activator, required for leukaemogenesis^66^. The small molecule inhibitor Celastrol, a triterpenoid, was used to disrupt the MYB/p300 interaction, therefore interrupting MYB signalling in leukaemic cells. Celastrol did not change *MYB* expression but inhibited the interaction of the transactivation domain of MYB with the KIX domain of p300. Accordingly, Celastrol strongly inhibited MYB-dependent transcriptional activation of target genes. Celastrol enhanced survival of mice transplanted with patient-derived HoxA9/Meis1-driven AML, confirming that targeting MYB transcription function could be an effective strategy in this leukaemia^67^. Another compound used to disrupt the interaction between MYB and p300, Naphthol AS-E phosphate, inhibited the expression of the *MYB* gene itself, as well as that of several MYB-target genes, inducing myeloid differentiation and apoptosis^68^. The negative effect of Naphthol AS-E phosphate on *MYB* gene expression could be a consequence of the block of *MYB* gene autoregulation. Nicolaides et al. showed that human *MYB* maintains high levels of its expression through an autoregulatory mechanism involving MYB-binding sites in the 5′ flanking region of the *MYB* gene itself^41^.

The anti-helminth agent mebendazole exhibited anticancer activity in AML human cell lines by interfering with MYB activity. Short-term exposure to the drug induced changes in the expression level of MYB-regulated genes in cells expressing the *MLL-AF9* fusion oncoprotein^69^. Expression of the MYB oncoprotein was drastically reduced in the presence of low concentrations of the drug in all cell lines analysed, whereas *MYB* mRNA levels were only reduced after exposure to very high mebendazole concentrations, and only in a few of the cell lines. This suggested that the drug acts mainly at the protein level. Indeed, inhibition of the proteasome reversed MYB protein loss, demonstrating that mebendazole causes proteasomal degradation of MYB by interfering with the heat shock protein 70 (HSP70) chaperone system. Importantly, mebendazole impaired AML cancer progression in vivo^69^.

5-hydroxy-2-methyl-1,4-naphthoquinone (also known as plumbagin) has been shown to target the transcriptional-activating domain (TAD) of MYB. By using the MYB TAD fused to the Gal4 DBD, the Klempnauer group observed that plumbagin inhibits transcription of a reporter gene containing GAL4-binding sites. Increasing the dosage of ectopically expressed p300, progressively antagonised the effect of plumbagin, demonstrating that the drug interfered with the p300–MYB interaction in AML cells^70^.

Recently, a peptidomimetic approach to block the activity of MYB was developed by designing an inhibitory peptide called MYBMIM. The MYBMIM inhibitory effect is caused by its ability to disrupt the MYB:CBP/p300 complex. MYBMIM directly binds to the KIX domain of CBP with an affinity similar to the naïve complex, causing its disassembly and reduced MYB*-*dependent expression of genes whose enhancers are occupied by it. NOD-scid mice engrafted with leukaemia cells treated with the peptide showed significant reduction of cancer burden, which was caused by mitochondrial apoptosis. Furthermore, ChIP analysis revealed a marked loss of the epigenetic mark H3K27ac on super-enhancers regulated by acetylation driven by p300:CBP, and consequent reduced expression of key MYB-regulated genes such as *MYC* and *BCL2*^71^.

##### *MYB* and paediatric low-grade gliomas (PLGGs)

PLGGs typically present gene fusions, especially related to component of the MAPK pathway, such as *BRAF*^72^. *MYB* rearrangements have been recently discovered in the context of whole-genome sequencing (WGS) and/or RNA-sequencing (RNA-seq) of 249 samples of PLGGs, leading to the identification of recurrent *MYB-QKI* fusions in angiocentric gliomas^73^. MYB fused to the RNA-binding protein QKI confers oncogenic properties using three distinct mechanisms. Firstly, the alteration results in the translocation of a super enhancer located in the 3′ untranslated region of QKI upstream the *MYB* promoter, resulting in its activation. Secondly, the MYB-QKI fusion protein acts as transcription factor, binding and activating the *MYB* promoter through a positive feedback loop. Thirdly, hemizygous loss of *QKI* expression caused by the rearrangement of its locus contributes to oncogenesis since it functions as a tumour-suppressor gene^74–76^. Gene-set enrichment analysis (GSEA) revealed that the expression of *MYB-QKI* fusion was associated with *MYB* signature genes^73^. MYB protein structure and its modifications found in tumours are fundamental for its transforming ability. In fact, as already mentioned above, full-length MYB is not endowed with a strong oncogenic activity in vitro, whereas C-terminal truncations are required for its activation^77^. *MYB-QKI* breakpoints in *MYB* intron 9–15 result in C-terminal truncation and oncogenic activation of *MYB*^73^.

##### *MYB* and cancers of the gastrointestinal tract

80% of colorectal cancers are characterised by *MYB* overexpression, which is associated with tumour aggressiveness and poor prognosis^78,79^. *MYB* overexpression in colon cancer is a consequence of mutations in intron 1 regulatory sequence^80^. Given the broad presence of the oncoprotein in this cancer, investigators in the Australian Peter MacCallum Cancer Centre engineered a vaccine against the MYB antigen called TetMYB. It is composed of an inactivated MYB protein flanked by the tetanus toxin T cell epitopes cloned into the pVAX1 plasmid vector. The immunotherapeutic role of the pVAX1-Tet-human MYB DNA vaccine was investigated in colon and adenoid cystic carcinoma (ACC) patients, also in combination with the anti-PD-1 antibody BGB-A317 to assess safety and maximum tolerated dose (MTD) in a first-in-human clinical trial^81^. This approach should overcome limitations caused by epitope/MHC restriction when targeting an endogenous antigen, as its application will not depend upon a need to match the patient’s MHC subtype. The trial, if successful, could pave the way for vaccine treatment not only of colorectal cancers or ACC, but also other *MYB*-expressing cancers. This clinical trial is based upon preclinical studies of the same Australian group in mice transplanted with MC38 colon adenocarcinoma cells expressing high levels of *MYB*. Breaking peripheral tolerance with the vaccine strategy enhanced anti-tumour immunity mediated by both CD4+ and CD8+ T cells, without insurgence of autoimmunity, causing a significant suppression of MC38 cancer growth^78^. *MYB* alterations have been also observed in pancreatic cancer, where it has been shown to interact with genes required for proliferation, survival and metastasis^82^.

##### *MYB* and breast cancer

MYB has been found bound to more than 10,000 promoters in MCF-7 breast cancer cells and recognised as a key activator of downstream targets, including genes involved in cancer progression and metastasis, such as cyclooxygenase-2 (*COX-2*), *BCL2*, *BCLXL*_,_ *JUN*, *KLF4*, *NANOG*, *MYC*, and *CXCR4*^19^. Breast cancer is a heterogeneous disease with a clinical outcome strictly determined by molecular profiles^83,84^. Over 70% of human breast cancers are oestrogen receptor-positive (ER+) and express *MYB*^85^. Gonda and colleagues reported for the first time that inhibition of *MYB* expression severely impairs the proliferation of ER+, but not ER−, breast cancer cell lines^37^. The relationship between *MYB* and ER is also indicated by the expression of *MYB* in normal, ER+ murine mammary epithelial cells, suggesting a salient role of the MYB transcription factor in mammary cell proliferation and tumour development in the human and mouse systems^37,86^.

ER+ breast cancer benefits from endocrine therapy (ET), which can reduce local and distant cancer recurrence and mortality rate^87,88^. ET can be administrated as neoadjuvant, adjuvant or palliative treatment and includes aromatase inhibitors, selective ER modulators (SERMs) such as tamoxifen, and antagonists such as fulvestrant^89^.

In ER+ve breast cancer patients, *MYB* expression is oestrogen-dependent, since it was observed that *MYB* mRNA levels were 5-fold higher 24 h after stimulating breast cancer cells with beta-estradiol, suggesting a strong correlation between the proto-oncogene expression and ER status in cancer^19^. *MYB* expression in ER+ve breast cancer cells is regulated at the level of transcriptional elongation, leading to the hypothesis that CDK9 inhibitors could be used to indirectly target *MYB* in this cancer. Indeed, CDK9 inhibition resulted in apoptotic death of breast cancer cell lines, accompanied by dose-dependent inhibition of the *MCL-1* gene and protein expression^90^. CDK9 inhibitors also impaired cell proliferation and cell cycle progression, inducing arrest at both the G1/S and G2/M phases of the cell cycle. Moreover, this led to the downregulation of MYB target genes involved in cell cycle progression such as *CCNB1* and *CCNE1*, which was reversed by ectopic expression of *MYB*^90^.

Breast cancer patients often develop resistance to treatment. Activation of epithelial–mesenchymal transition (EMT) is a mechanism by which breast cancer cells acquire resistance to targeted therapies^91^. Micro-RNAs have been implicated in the EMT process, particularly the miR-200 family^92,93^. Following ectopic over expression of miR-200b/c in drug-resistant cells, *MYB* expression levels decreased, indicating that it is a target of miR-200s. After silencing *MYB* in an ER+ve breast cancer cell line refractory to tamoxifen therapy, the authors of the study observed that the EMT markers vimentin, ZEB1, and ZEB2 were downregulated, further supporting the hypothesis that *MYB* is involved in EMT and drug resistance in breast cancer. Indeed, as expected, breast cell line sensitivity to tamoxifen therapy was increased after inhibiting *MYB* expression^94^.

##### *MYB* and ACC

**Fig. 2:**
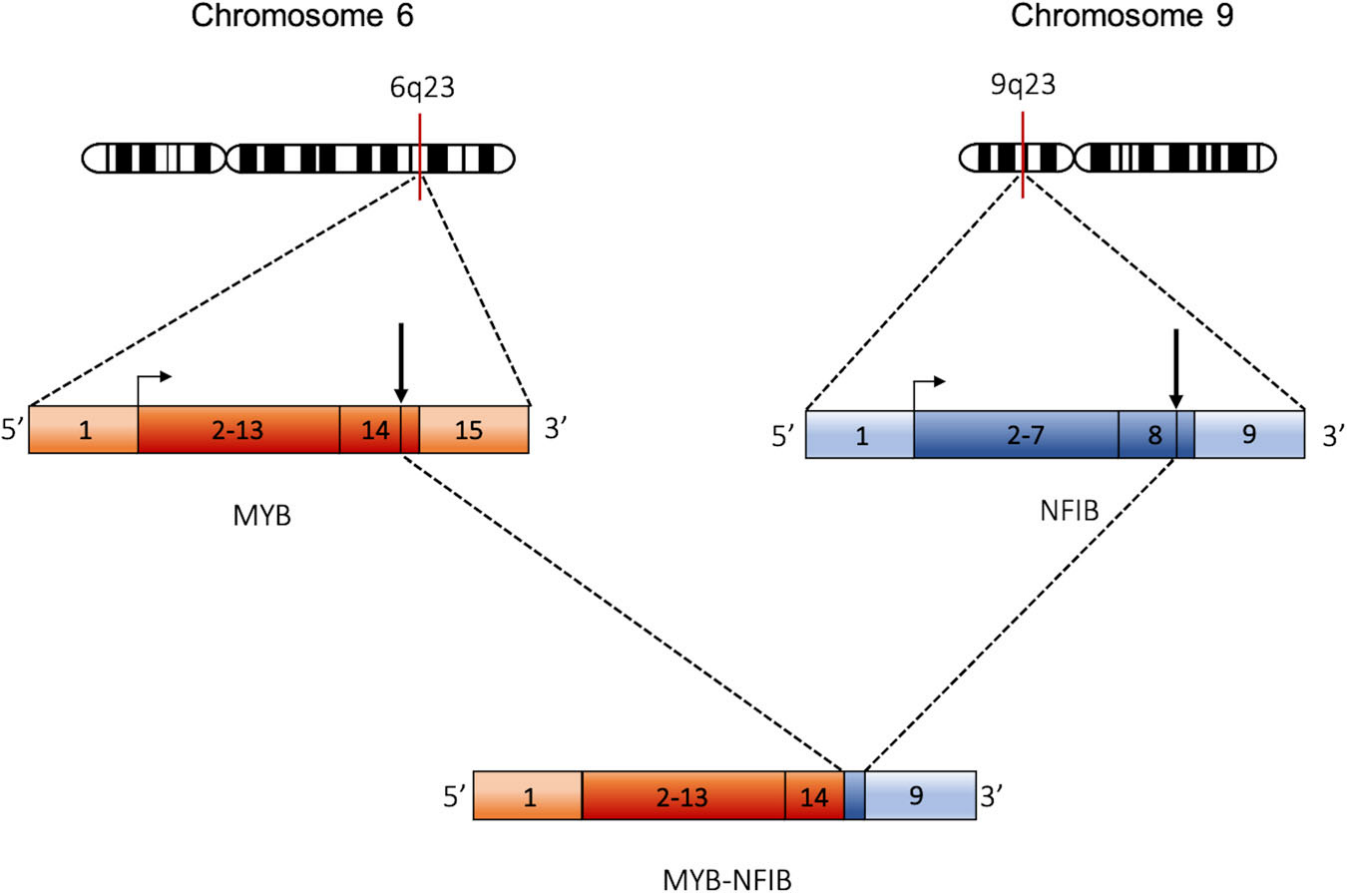
Schematic illustration of the MYB-NFIB fusion gene. The t(6;9) translocation results in a *MYB-NFIB* gene fusion. Arrows indicate the breaking points.

Stenman and colleagues discovered the translocation t(6;9)(q23;p23) as a genomic hallmark of ACC^95^. The translocation results in the fusion of the carboxyl-terminus of the MYB oncoprotein to five amino acids (SWYLG) encoded by the last exon of *NFIB* (Fig. 2)^95,96^. ACC is characterised by the presence of the *MYB*-*NFIB* fusion gene in 30–86% of cases, depending on the study^97,98^. An important consequence of chromosomal rearrangements in ACC is the translocation of strong enhancers near the *MYB*, or *MYBL1*, locus, which activates their transcription^99^. Rearrangements of the *MYB* locus have been observed in ACCs of the breast, lungs or glands in different body locations and in cylindromas, suggesting that *MYB* activation is frequent in exocrine gland tumours^98,100–102^. Another consequence of the chromosomal translocations detected in ACC and other gland tumours is, in some cases, loss of genetic material. In this regard, Mitani and colleagues theorised that two genetic events drive ACC pathogenesis: one involves the generation of fusion genes resulting from reciprocal translocation between chromosome 6q and 9p or other partners, and the other event constitutes a loss of genetic material, denoting the presence of one or more tumour suppressor genes^103^. Most ACCs do not acquire a large number of genetic changes, typical of other carcinomas^104^. Over half of ACC cases present chromosome 6 deletions, suggesting an important selection for these alterations in the molecular aetiology of these neoplasms. However, efforts to identify a tumour suppressor gene at these loci in ACC have been unsuccessful to date^103,105^.

MYB-NFIB is a putative oncoprotein, which has been shown to control ACC tumour cell proliferation and spherogenesis^106^. Intriguingly, the fusion gene is regulated by AKT-dependent signalling downstream of the IGF1 receptor and its expression can be downregulated by IGF1R-inhibition with linsitinib. Furthermore, EGFR and MET signalling also promote growth of ACC cells^106^. In line with these findings, evidence in patients or xenograft models indicate that monoclonal antibodies targeting IGF1 or EGF receptors could be effective drugs in ACCs expressing the fusion oncoprotein^107–109^. To investigate the implication of the *MYB-NFIB* fusion gene in ACC, Mitani and co-workers analysed a cohort of 123 salivary carcinomas, including primary ACCs of the salivary gland, metastatic ACCs, non-ACC salivary carcinomas, and normal salivary gland tissues^103^. Using RT-PCR, validated by fluorescence in situ hybridisation (FISH) analysis, they found that among 89 ACC cases (72 primary ACCs and 17 metastatic), 26 were positive for expression of the *MYB-NFIB* fusion transcript. Interestingly, none of the 34 non-ACC carcinomas were positive. In addition, 14 different fusion transcripts involving multiple exons of *MYB* and *NFIB* were identified. To provide further insights on the role of *MYB* in this cancer, expression of the wild type or fusion *MYB* transcripts was quantified. Unsurprisingly, *MYB* expression was elevated in *MYB-NFIB* fusion positive ACCs, probably caused by loss of the negative regulatory sequence at the 3′ untranslated region of *MYB*. Interestingly, the expression of wild type *MYB* was elevated >40-fold in fusion-negative ACCs compared to non-ACC carcinomas, and only 2-fold lower than fusion-positive ACCs. The authors concluded that whereas genomic rearrangement must be causative of *MYB* overexpression in fusion positive ACCs, alternative mechanisms may be responsible for *MYB* overexpression in fusion negative ACCs^103^. Thus, *MYB* overexpression is a frequent consequence of the *MYB-NFIB* fusion in glandular tumours, but can also occur via other mechanisms.

The polyether ionophore monensin was recently identified as a *MYB* inhibitor using a luciferase-based screen and tested on ACC cell lines derived from ACC patients. These cells were more sensitive to the anti-cancer agent than *MYB*-expression negative, control cell lines. Monensin suppressed both *MYB-NFIB* mRNA and protein levels. Moreover, the compound, and related polyether ionophores, also induced differentiation and promoted apoptosis of leukaemic cell lines, suggesting that *MYB* inhibitors can be effective against solid and liquid malignancies^110^. Using a chemical screen in Zebrafish, the group of Leonard Zon have demonstrated that retinoic acid is a suppressor of MYB in ACC. All trans retinoic acid (ATRA) treatment of mice bearing patient-derived ACC tumours showed reduced expression of *MYB* and binding of MYB at translocated enhancers. Importantly, ATRA inhibited the expression of cell cycle related, MYB-target genes. ATRA is used in the clinic for the treatment of promyelocytic leukaemia and has a known safety profile, suggesting that it will be soon used in the context of a clinical trial in ACC patients^111^.

Identification of actionable target genes downstream of *MYB* can be a reasonable alternative to avoid negative consequences caused by inactivation of the wild-type MYB transcription factor. Indeed, the potential haematologic toxicity of anti-MYB therapies could be further exacerbated in patients under regimens of chemotherapy and radiotherapy. An important gene axis regulated by MYB is the insulin growth factor and its receptor. Interestingly, insulin growth factor receptor (IGFR) signalling positively regulates *MYB-NFIB* in ACC, suggesting that *MYB* and *IGFR* are engaged in a feed forward loop in cancer^112,113^. Accordingly, it has been shown that the small molecule inhibitor Linsitinib or the therapeutic antibody Figitumumab reduce the growth of ACC tumours in mouse models and in patients, suggesting that targeting IGFR signalling could be an effective strategy in *MYB* overexpressing cancers^109,112^. In an effort to identify new MYB target genes in ACC, our group has generated retroviral vectors expressing wild-type *MYB* or two *MYB-NFIB* variants derived from ACC patients. The different *MYB* isoforms were ectopically expressed in immortalised breast MCF10A cells, and genes up or downregulated were identified by microarrays. GSEA revealed that *ATR/BRCA* was the top activated downstream pathway, with a significant upregulation of *ATR* gene expression^43^. *ATR* mRNA levels were increased in primary ACCs compared to normal salivary glands. Accordingly, the clinical ATR kinase inhibitor VX-970 caused apoptosis of primary ACC cells in vitro and significant shrinkage of ACC patient-derived xenografts. These results support the theory that acting on downstream target genes/proteins might be a worthy—and even safer—alternative to directly targeting the *MYB* gene itself^43^.

Surgery is the first line treatment for ACC, followed by cytotoxic chemotherapy and/or radiotherapy as adjuvant treatments to avoid recurrence. Unfortunately, standard treatments only provide limited benefit in advanced disease, which is usually lethal, with a high rate of recurrence and metastasis. Therefore, new and more effective treatments are urgently needed for these high-risk patients. Previous clinical trials have led to the approval of tyrosine kinase inhibitors (TKI) for the treatment of aggressive forms of solid malignancies, such as thyroid cancer refractory to radio therapy and unresectable hepatocellular carcinoma^114,115^. Most of the targeted tyrosine kinases are also MYB regulated, such as vascular endothelial growth factor receptors (VEGFRs), fibroblast growth factor receptors (FGFRs), the stem cell factor receptor KIT (c-KIT), FMS-like tyrosine kinase 3 (FLT3), platelet-derived growth factor receptors (PDGFRs), and the proto-oncogene RET^95,115–119^. Persson and co-workers have recently shown that *VEGFA*, *FGF2*, *KIT* and other genes encoding receptor tyrosine kinases are commonly overexpressed in ACC samples, leading to consider TKIs as credible candidates for the treatment of relapsed/metastatic ACC patients^95^. However, it has been observed an overall poor response in therapies against these targets in ACC, suggesting that other, more relevant *MYB* downstream genes should be clinically exploited in this tumour.

### *MYBL1*

*MYBL1* is predominantly expressed in the central nervous system (CNS), germinal B-lymphocytes, mammary gland ductal epithelium, and in the testis^120,121^. It has a key role in spermatogenesis, particularly in cell cycle progression of germ cells through pachynema^121,122^. *MYBL1-null mice* are viable, but exhibit growth abnormalities as well as defects in spermatogenesis and female breast development^120^.

#### *MYBL1* alterations in cancer

*MYBL1* rearrangements are a hallmark of low-grade gliomas (LGGs), the commonest paediatric CNS neoplasm, arising in children and adolescents^114,123^. Recent molecular characterisations through WGS have led to the identification of new genetic alterations in LGGs. These studies have identified activation of the MAPK/ERK pathway caused by the duplication of the tyrosine kinase domain (TKD) of the *FGFR1* gene and frequent rearrangements of the *MYB* family members *MYB* and *MYBL1* in diffuse cerebral LGGs^124^. 8q13.1 gain was observed as a significant recurrent event in diffuse astrocytoma grade IIs. This leads to a duplication of *MYBL1* and truncation of its C-terminal NRD, resulting in anchorage-independent growth of NIH-3T3 cells and tumour formation in nude mice^125^. *MYBL1* gene amplification is a distinct alteration of the subtype IDH-wt/H3-wt of diffuse gliomas, together with *TERT* and *BRAF* mutations, *EGFR* and *FGFR1* alterations, and other chromosomal aberrations^126^. Although these alterations are rare, sequencing analysis of uncommon low-grade neuro-epithelial tumours revealed that these pathogenic mutations occur at a high frequency (78%) in this cohort^114^.

Patients with isomorphic diffuse glioma or astrocytoma can harbour copy number alterations of *MYBL1* or *MYB* (13 out of 25 samples, 52%), as assessed with RNA sequencing. Gene fusions accounted for 50% of cases^127^.

ACC is characterised by the chromosomal translocation t(6;9), leading to the expression of the *MYB-NFIB* fusion gene^95^. Although *MYB* is the *MYB* family member most often involved in this cancer, it was recently demonstrated that a subset of ACCs contains the t(8;9) chromosomal translocation^128^. This results in the creation of a *MYBL1-NFIB* gene fusion, which probably functions in a manner similar to *MYB-NFIB*, given the structural analogies between MYBL1 and MYB. Indeed, tumours with *MYB* and *MYBL1* translocations display overlapping gene expression profiles and clinical outcome, suggesting that the related MYB proteins are interchangeable oncogenic drivers in ACC. The research group that identified the translocation t(8;9), also highlighted a t(8;14) translocation, leading to the fusion of *MYBL1* to the *RAD51B* gene^128^.

In *MYB* or *MYB-NFIB* negative subsets of breast ACC tumours, alternative genetic mechanisms of *MYB* activation have been demonstrated. RNA and WGS unveiled that these cancers could harbour *MYBL1* rearrangements, including those between *MYBL1-ACTN1* and *MYBL1-NFIB*^102^. In these rare triple negative breast cancers (TNBC), the histological pattern was identical to the *MYB-NFIB*-positive, salivary gland ACCs. The *MYBL1* rearrangements were confirmed at genomic level by the FISH technique. The translocation results in an in-frame chimeric transcript containing the DNA-binding and transactivating domains, encoded by exons 1–14, of *MYBL1* fused to the exon 9 of *NFIB*. In addition, another in-frame fusion between *MYBL1-ACTN1* was also detected for the first time in ACC samples. The fusion leads to loss of the C-terminus region of MYBL1 due to the fusion of exons 1–8 of *MYBL1* with exons 10–21 of *ACTN1*^102^.

Another organ in which ACC neoplasms can originate is the lung. Primary tracheobronchial ACC is one of the rarest types of lung cancer, accounting for <1% of cases. Pei et al. analysed 7 lung ACCs, documenting that 7 out of 7 cases presented *MYB* or *MYBL1* genes fused with *NFIB* or, less frequently, with *RAD51B*^101^. Primary cutaneous ACCs display a genetic landscape similar to those of salivary glands, showing fusions of either *MYB* or *MYBL1* with the common partner *NFIB*^129^.

### *MYBL2*

*MYBL2*, encoding the transcription factor MYBL2, is ubiquitous and often co-expressed with other *MYB* members. It has been shown to regulate cell cycle progression, cell survival and differentiation being an essential component of the DREAM complex^130–133^. It is also a promoter of cell survival by activating antiapoptotic genes such as *BIRC5* (survivin), *CLU* (ApoJ/clusterin) and *BCL2*^134–137^. MYBL2 has been shown to aid repair of DNA double-strand breaks, supporting genome stability in haematopoietic and pluripotent stem cells^138,139^. Expression of *MYBL2* is important for both normal and transformed cell homoeostasis. This concept is supported by the early embryonic lethal phenotype of *MYBL2* knockout mice, due to impaired inner cell mass formation, or suppression of cell cycle progression and cell survival in oesophageal, hepatic, colorectal, and sympathetic nervous system cancer cells in which the expression of *MYBL2* has been downregulated^140–145^. The activity of *MYBL2* is highly regulated at transcriptional and post-transcriptional levels. Cyclins and their catalytic partners, the cyclin-dependent kinases (Cdks), function as key regulators of the cell cycle^146^. Cyclin D1 with Cdk4 or Cdk6 has been shown to play an important role at the ‘restriction point’ in the G1 phase of the cell cycle before cells enter into the mitotic cycle, whereas, for the transition from G1 to S phase, cyclin E–Cdk2 complexes are the most critical, and cyclin A–Cdk2 complexes are required during S phase^146^. *MYBL2* is regulated by the transcription factor E2F and required for the expression of cyclin B and cdc2 in G2/M^147–149^. When overexpressed, the tumour suppressor protein p53 induces Waf1/Cip1/p21 protein-dependent cell-cycle arrest and activation of MYBL2 allows cells to escape this block, suggesting that MYBL2 acts at a later stage than Waf1/Cip1/p21 during cell-cycle progression^150,151^. MYBL2 is a substrate for cyclin A/E–Cdk2 kinase activity and its transcriptional activity is regulated by phosphorylation^148,150^.

Genes involved in the G2/M phase of the cell cycle are activated by MYBL2 switching from the repressive DREAM to the MuvB (MMB) complex^136,152,153^. *MYBL2* is transcriptionally repressed in G1, activated by cyclin A/Cdk2-mediated phosphorylation during S-phase, and subsequently degraded in late G2 in a ubiquitin-dependent manner^147,148,154,155^. Phosphorylation of MYBL2 occurs at Serine or Threonine residues followed by Proline^156^. Pin1 isomerase recognises the pSer/pThr-Pro residues altering functions of the MYBL2 protein by inducing conformation changes. Cdk-dependent phosphorylation and Pin1 isomerization induce Plk1 kinase binding to MYBL2. Plk1 phosphorylates the region of MYBL2 containing the transcriptional activation domain (TAD), suggesting that PLK1-induced modification of MYBL2 is crucially required for transcriptional activation of pro-mitotic genes^157^. Consistent with an important role in cell cycle progression, down-regulation of *MYBL2* leads to spindle and centrosome defects, arrest in the G2/M phase of the cell cycle, failure in cytokinesis, polyploidy and apoptosis^132,158^.

#### *MYBL2* alterations in cancer

The DREAM complex [DP, RB-like, E2F4 and MuvB (synMuv genes, class B)] is a master coordinator of cell cycle-dependent gene expression and the balance between repressive DREAM and activating MYB-MuvB (MMB) complexes is frequently perturbed in cancer^159–162^. Increased expression of several components of the MMB complex, including MYBL2 and FOXM1, correlates with aggressive tumour features and poor prognosis^144,163^. To investigate the clinical relevance of the MYBL2/FOXM1/CDK/PLK1 axis, Werwein et al. used a pan-cancer resource of expression signatures that correlate cancer gene expression and clinical prognosis data, called PRECOG^157^. Interestingly, among the 50 genes (and their products) analysed, 44 (including *MYBL2*, *FOXM1*, *CCNA2*, and *PLK1*) were found to be targets of DREAM-mediated repression, while 29 of them were also targets of MMB activation^157^. Overexpression of *MYBL2* disturbs myeloid differentiation and promotes the progression of solid cancers where it is also an indicator of poor prognosis^133,142,164–166^. *MYBL2* is frequently overexpressed in malignancies including breast cancer, non-small-cell lung cancer (NSCLC), AML, colorectal cancer, pancreatic ductal adenocarcinoma, and neuroblastoma^167–175^.

The molecular mechanisms causing increased expression of *MYBL2* in multiple human cancers are still not fully elucidated.

##### *MYBL2* and breast cancer

Alterations of gene expression might be caused by amplification of the *MYBL2* locus located in chromosome 20q13^176–178^. 20q13 amplification or copy gains are common in breast cancer and are usually associated with poor prognosis^176^. Notably, a *MYBL2* germline polymorphism causing the Serine-to-Glycine amino acid change S427G is correlated to a high risk of basal-like breast cancer^133^. Moreover, *MYBL2* overexpression was noted in HER2+/ER− and luminal B breast cancer samples, but not in luminal A or normal breast tissue, strongly suggesting a correlation between *MYBL2* expression and aggressiveness of breast cancer^177^. A recent review explains the molecular mechanisms of *MYBL2* amplification and new therapeutic opportunities in breast cancers^179^.

##### *MYBL2*, clear cell renal cell carcinoma (ccRCC) and NSCLC

ccRCC is the most frequent renal malignancy^180^. *MYBL2* expression could be used as a biomarker to predict patients’ prognosis in this cancer. *MYBL2* was found upregulated in a cohort of 530 ccRCC patients compared to healthy tissues. Of note, upregulated *MYBL2* was significantly associated with age and sex of cancer patients, advanced T stage, lymph node and distant metastases, clinical stage and histological grade^181^. Moreover, a significant correlation between high *MYBL2* expression and worse prognosis was established by Kaplan–Meier analysis, indicating that *MYBL2* expression is an independent biomarker of progression in ccRCC^181^.

Another neoplasia characterised by deregulation of *MYBL2* is non-small-cell lung cancer (NSCLC). Analysis of MCODE clusters highlighted genes involved in “driver networks” for NSCLC, which include the transcription factors FOXM1, TFDP1, E2F4, SIN3, and MYBL2^182^. A further study confirmed the potential oncogenic role of *MYBL2* in NSCLC. Through chromatin immunoprecipitation (ChIP) assay, researchers identified a direct binding between MYBL2 and the gene *Non-SMC CondensinIComplex Subunit H* (*NCAPH*), well-known to have oncogenic properties in lung cancer. A significant correlation between high *NCAPH* expression and poor prognosis was confirmed, suggesting that targeting the MYBL2-regulated gene could be of potential therapeutic value in this setting^169^.

##### *MYBL2* and leukaemia

*MYBL2* overexpression is a prognostic factor in AML, defining a subset of patients with poor prognosis^170,183^. This could be linked to the reduced expression of miR-30a, miR-30b and miR-30c, involved in the regulation of haematopoiesis and cell differentiation, which were shown to be expressed at lower levels in *MYBL2*^*high*^ AML samples^170,184,185^. The strong correlation between overexpression of *MYBL2* and downregulation of the miR-30 cluster suggests that the micro-RNAs antagonise the expression of *MYBL2*, or that the latter suppresses miRNAs expression in AML^170^.

A recent study published in *Cell* revealed a link between MYBL2 and the protein phosphatase 2A (PP2A) in leukaemia. Morita and co-workers identified a class of small molecule that they called iHAPs—improved heterocyclic activators of PP2A—able to activate a PP2A complex, which suppresses tumour progression. PP2A is an enzyme formed by different subunits; among them, PPP2R1A, PPP2CA, and PPP2R5E are strictly required for antitumor activity^186^. Using isotope-labelled amino acids (SILAC) and mass spectrometry analysis, substrates dephosphorylated by PP2A in the presence or absence of iHAPs were identified, among which MYBL2. The researchers were able to activate the PP2A complex, usually present in an inactive form in cancers due to the overexpression of inhibitory proteins, and observe dephosphorylation of MYBL2 on Ser241, required for transactivation of cell cycle-related genes, resulting in an irreversible growth arrest of multiple cancer cells. Thus, MYBL2 is centrally involved in cancer cell proliferation and can be indirectly targeted by small molecule-mediated reactivation of the PP2A tumour suppressor protein^186^.

## Conclusions

The MYB transcription factors are a point of convergence of numerous signalling pathways essential for multiple cellular functions, and their deregulation has been associated with aggressive behaviour of cancer cells. Reflecting the high similarity of protein structures, MYBL1, MYBL2, and MYB are all involved in the control of cell survival, proliferation and differentiation. One could hypothesise that spatio-temporal differences in gene expression during organogenesis and in pathological conditions may determine specific MYB requirements in cells. The ever-expanding number of studies reporting deregulation of MYB family members in the pathogenesis of human cancers is instigating researchers to find new and more efficient methods to target these transcription factors. Direct pharmacological inhibition of *MYB* or its product MYB, is emerging as a potential therapeutic strategy for both liquid and solid malignancies. Nevertheless, inhibiting MYB could potentially lead to haematopoietic toxicity, indicating that targeting downstream target genes and coactivator molecules might make more clinical sense. Further studies will be required to develop effective therapeutic interventions aiming at suppressing MYB signalling in tumours while minimising risks to patients.
